# Beyond T2-FLAIR mismatch sign in isocitrate dehydrogenase mutant 1p19q non-codeleted astrocytoma: Analysis of tumor core and evolution with multiparametric magnetic resonance imaging

**DOI:** 10.1093/noajnl/vdae065

**Published:** 2024-07-18

**Authors:** Jian Ping Jen, Xuanxuan Li, Markand Patel, Huzaifah Haq, Ute Pohl, Santhosh Nagaraju, Victoria Wykes, Paul Sanghera, Colin Watts, Vijay Sawlani

**Affiliations:** Department of Neuroradiology, University Hospitals Birmingham, Birmingham, UK; Department of Radiology, Huashan Hospital, Fudan University, Shanghai, China; Department of Neuroradiology, University Hospitals Birmingham, Birmingham, UK; Department of Neuroradiology, University Hospitals Birmingham, Birmingham, UK; Department of Cellular Pathology, University Hospitals Birmingham, Birmingham, UK; Department of Cellular Pathology, University Hospitals Birmingham, Birmingham, UK; Neuroimaging, University of Birmingham, Birmingham, UK; Department of Neurosurgery, University Hospitals Birmingham, Birmingham, UK; Neuroimaging, University of Birmingham, Birmingham, UK; Neuroimaging, University of Birmingham, Birmingham, UK; Department of Neurosurgery, University Hospitals Birmingham, Birmingham, UK; Department of Neuroradiology, University Hospitals Birmingham, Birmingham, UK; Neuroimaging, University of Birmingham, Birmingham, UK

**Keywords:** 1p19q non-codeleted astrocytoma, IDH mutant, intratumoral heterogeneity, spectroscopy, T2-FLAIR mismatch

## Abstract

**Background:**

The T2-FLAIR mismatch sign is an imaging correlate for isocitrate dehydrogenase (IDH)-mutant 1p19q non-codeleted astrocytomas. However, it is only seen in a part of the cases at certain stages. Many of the tumors likely lose T2 homogeneity as they grow in size, and become heterogenous. The aim of this study was to investigate the timecourse of T2-FLAIR mismatch sign, and assess intratumoral heterogeneity using multiparametric magnetic resonance imaging techniques.

**Methods:**

A total of 128 IDH-mutant gliomas were retrospectively analyzed. Observers blinded to molecular status used strict criteria to select T2-FLAIR mismatch astrocytomas. Pre-biopsy and follow-up standard structural sequences of T2, FLAIR and apparent diffusion coefficient, MR spectroscopy (both single- and multi-voxel techniques), and DSC perfusion were observed.

**Results:**

Nine T2-FLAIR mismatch astrocytomas were identified. 7 had MR spectroscopy and perfusion data. The smallest astrocytomas began as rounded T2 homogeneous lesions without FLAIR suppression, and developed T2-FLAIR mismatch during follow-up with falls in NAA and raised Cho/Cr ratio. Larger tumors at baseline with T2-FLAIR mismatch signs developed intratumoral heterogeneity, and showed elevated Cho/Cr ratio and raised relative cerebral blood volume (rCBV). The highest levels of intratumoral Cho/Cr and rCBV changes were located within the tumor core, and this area signifies the progression of the tumors toward high grade.

**Conclusions:**

T2-FLAIR mismatch sign is seen at a specific stage in the development of astrocytoma. By assessing the subsequent heterogeneity, MR spectroscopy and perfusion imaging are able to predict the progression of the tumor towards high grade, thereby can assist targeting for biopsy and selective debulking.

## Lay Summary

A particular type of brain tumor, known as IDH-mutant 1p19q non-codeleted astrocytoma, can show up bright on some magnetic resonance imaging (MRI) sequences (T2) but dark on others (FLAIR). Although these tumors often look uniform, sometimes there are irregular internal appearances, hinting at differences in cells within the tumor. The authors of this study aimed to understand the importance of these irregularities in imaging. To do this, they used advanced brain imaging techniques to study the tumor’s metabolism (MR spectroscopy) and blood flow (perfusion). Their results showed that the most abnormal regions of the tumor, in terms of both structure and metabolism, were located within the core of the tumor rather than around the edges.

**Key Points**

1. T2-FLAIR mismatch sign is absent initially, but develops with tumor maturation.
2. Many tumors become heterogenous as they grow. MR spectroscopy and perfusion can assess the heterogeneity.
3. The findings enable early prediction of transformation, and facilitate appropriate treatment.

**Importance of the Study**

This study makes 2 key points in a small subset of T2-FLAIR mismatch astrocytomas. Firstly, the presence of T2-FLAIR mismatch sign is a dynamic phenomenon, therefore the application of strict criteria is primed to accurately detect astrocytomas at a specific, stage in maturation. Following detection at this stage, T2-FLAIR astrocytomas may expand and/or become more internally heterogeneous as they evolve. Secondly, this intratumoral heterogeneity on structural imaging correlates with aberrations in MR spectroscopy and perfusion. The utilization of advanced magnetic resonance imaging techniques to assess tumor heterogeneity provides valuable imaging guidance for more accurate target for biopsy and aids in precise and selective resection, in case total resection is not possible.

The 2021 WHO glioma classification emphasizes the molecular markers isocitrate dehydrogenase (IDH), alpha-thalassemia mental retardation X-linked (ATRX), and codeletions of both the short arm of chromosome 1 (1p) and long arm of chromosome 19 (19q), or 1p19q codeletion, alongside traditional histology, to establish integrated diagnoses of astrocytoma, oligodendroglioma, or glioblastoma.^1^

The T2-FLAIR mismatch sign was originally described in 2017 and strongly correlated with the diagnosis of IDH mutant 1p19q non-codeleted gliomas (astrocytomas).^2^ The sign included two observations from standard magnetic resonance imaging (MRI): (1) complete or near-complete and almost homogeneous hyperintense signal on T2-weighted images, with (2). relatively hypointense signal on T2-weighted FLAIR (or FLAIR suppression), except for a hyperintense peripheral rim. This sign has a pooled sensitivity of 40% and specificity of 97% for astrocytoma.^3^ T2-FLAIR mismatch is not seen in 1p19q-codeleted tumors (oligodendrogliomas). Numerous institutional and multicentre retrospective studies have since confirmed reproducibility of the T2-FLAIR mismatch sign for astrocytoma when strictly applied.^2,4–6^ However, false positives have been reported when criteria are loosely applied, in enhancing tumors, and in pediatric patients.^7–9^

T2-FLAIR mismatch is a specific morphological observation that can be made in the early tumor lifecycle, likely due to increased microcystic change within the tumor, which has been shown in direct sampling studies.^10,11^ Microcystic change is associated with ultra-long T1 and T2 relaxation times, read as FLAIR suppression which aims to suppress values from extremely long repetition times (TR).^12^

However, there remains subjectivity in interpretation of sequences in T2-FLAIR mismatch tumors. Our previous study showed that T2-FLAIR mismatch sign is only seen in a part of the non-enhancing IDH-mutant diffuse glioma cases at certain stages, and MRI techniques were able to further estimate the grading of astrocytomas with high accuracy.^13^ Many of the tumors likely lose T2 homogeneity as they grow in size, and become heterogenous. We aimed to investigate the time course of T2-FLAIR mismatch sign, and the mechanism of intratumoral heterogeneity using multiparametric MRI techniques and extended follow-up. We hypothesize that advanced MRI techniques are able to evaluate the heterogeneity distribution, and predict the potential grading of the tumors.

## Materials and Methods

Clinical data were collected with Institutional Review Board approval. CARMS number 16115. The need for ethical approval was waived off from the Institutional Review Board for this retrospective study.

### Search Technique

The histopathology database was searched for patients matching “IDH Mutant” AND “Astrocytoma” or “Oligodendroglioma” from 2014 to 2019.

### Imaging Review Technique

Following this, the earliest pre-biopsy index MRI and follow-up MRIs were reviewed by two primary reviewers blinded to molecular status: JP (Neuroradiology Registrar, 4 years of experience), MP (Neuroradiology Registrar, 7 years of experience). All positive and equivocal cases were resolved through discussion with VS (Neuroradiology Consultant with interest in Neuro-Oncology, > 20 years of experience). Specific morphological features were gathered including shape (rounded or spreading), T2/FLAIR margin (sharp or indistinct), and T2 signal (predominantly homogeneous or heterogeneous). T2-FLAIR mismatch astrocytomas were gathered using the criteria below and when possible, interrogated with multiparametric MRI.

### T2-FLAIR Mismatch Sign Criteria for 1p19q Non-codeleted Astrocytoma

- Non-enhancing.
- T2 near homogeneous hyperintensity.
- FLAIR central near complete suppression.
- FLAIR rim near complete hyperintensity.
- Exclusion criteria.
  ◦ Clearly enhancing tumors, i.e. not faint, punctate, or linear.
  ◦ Pediatric patients.
  ◦ Insufficient imaging, eg. no FLAIR, too degraded.

### Image Acquisition

Conventional pretreatment MRI included a heterogenous real-world distribution of studies which included a mixture of 1.5T and 3T scans, both internal and external, and a variety of sequences. Minimum required sequences were axial T2, axial or 3D FLAIR, and T1 pre- and post-gadolinium. Additional sequences included diffusion-weighted imaging and apparent diffusion coefficient (ADC) maps, MR spectroscopy, T2* dynamic susceptibility contrast-enhanced (DSC) perfusion, and diffusion tensor imaging.

Multiparametric imaging was acquired using a 3T scanner (Magnetom Verio; Siemens, Erlangen, Germany) and 32-channel phased-array head coil. DSC perfusion imaging was performed with gradient-echo echo-planar imaging (GE-EPI) during the first pass of a standard dose (7.5 mmol) bolus of gadolinium-based contrast agent (Gadovist, Bayer Schering Pharma, Berlin, Germany), administered intravenously at a flow rate of 6 mL/s. A total of 80 imaging volumes are acquired at a temporal resolution of 2.1 s with the bolus typically arriving between the 10th and 15th volume. This was followed by a post-contrast 3D T1-weighted (T1W) magnetization-prepared rapid acquisition with gradient echo sequence acquired in the axial plane with sagittal and coronal reformats.

MR spectroscopy was performed using a combination of multi-voxel (for tumoral regions) and single-voxel point resolved spectroscopy sequences (PRESS), with short echo (TE = 30 ms, or single-voxel spectroscopy (SVS) 30) and intermediate echo (TE = 135 ms, SVS 135). 2D MR spectroscopic chemical shift imaging (CSI) was performed in the axial plane after choosing the slice with the largest cross-sectional diameter of the tumor, or greatest heterogeneity on T2, FLAIR, or ADC maps. This was followed by SVS with placement of the volume of interest further guided by the metabolic profile estimated on CSI. The single-voxel method was then used. Information was combined from structural imaging and CSI to sample the most relevant part of the lesion likely to provide the highest quality spectrum. In our institution, cutoff values for high-grade features are defined as ADC < 1000, relative cerebral blood volume (rCBV) > 2, Choline/Creatine ratio (Cho/Cr) > 1.8.^14^

### Molecular Inclusion Criteria

The method of 1p19q status testing consisted of Fluorescent in situ hybridization on fixed formalin paraffin embedded sections cut at 2–3um depth using the Vysis 1p19q dual color, site-specific probe from Abbott Molecular.

- Astrocytoma:
  ◦ IDH mutant + 1p19q non-codeleted.
  ◦ OR IDH mutant + ATRX mutant + clear astrocytic histology, in the absence of 1p19q status^15^
- Oligodendroglioma: IDH mutant + 1p19q codeleted.
  ◦ ATRX mutation is not typically associated with oligodendroglioma.
- Exclusion: 1p19q partial deletions, 1p19q unknown + ATRX wild type, pre-2016 histology diagnoses with 1p19q unknown + ATRX unknown, IDH-wild type, histone mutants, inconclusive diagnoses.
*N.B. If initial IDH-1 testing was negative the presence of a non-canonical IDH mutation was always checked for before diagnosing IDH-wild type glioblastoma.

## Results

### T2-FLAIR Mismatch Sign

**Figure 1.**
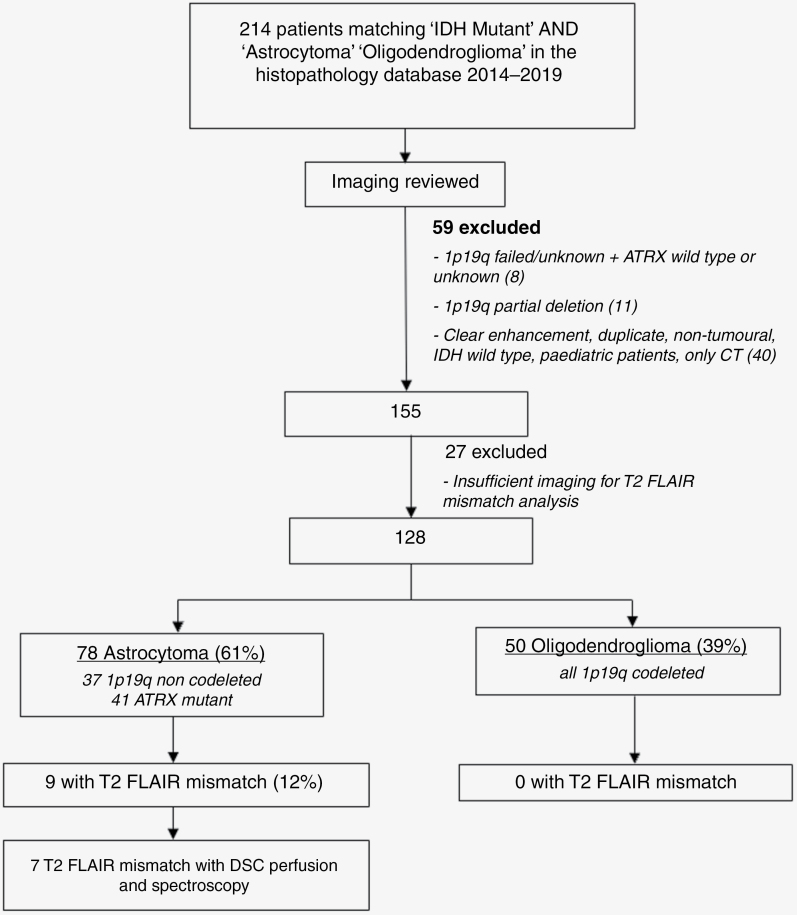
Flowchart of selection algorithm.

Of the 214 patients assessed, 128 non-enhancing IDH mutant tumors were included in the final analysis, 78 astrocytomas (61%) and 50 oligodendrogliomas (39%). 9 showed T2-FLAIR mismatch, all IDH mutant ATRX mutated tumors with clear astrocytic histology or 1p19q non-codeletion. These were all confirmed astrocytomas, according to the molecular inclusion criteira described above: 3/9 were male (33%), 6/9 female (67%), mean age of 35 ± 13 years. Test sensitivity was 12% and specificity 100% for 1p19q non-codeleted or ATRX mutant astrocytoma (Figure 1).

### Basic T2/FLAIR Structural Imaging of Astrocytomas and Oligodendrogliomas

A total of 24% (19/78) of astrocytomas were predominantly T2 homogeneous compared to only 6% (3/50) of oligodendrogliomas, χ2: 7.09 (*p* = 0.008), and the remaining lesions were T2 heterogeneous. All T2 homogeneous astrocytomas were rounded and sharply marginated. Of these, 47% (9/19) showed T2-FLAIR mismatch, 32% (6/19) were excluded due to a very strict application of criteria, and the remaining 21% (4/19) did not show central FLAIR suppression. Among oligodendrogliomas, rounded shape, and T2 homogeneous manifestations were very unusual. The 3 positive cases were small at presentation, and either showed a subtle infiltrative T2/FLAIR border at presentation or earlier on follow-up. None showed T2-FLAIR mismatch.

### Multiparametric Imaging of T2-FLAIR Mismatch Tumors

**Figure 2.**
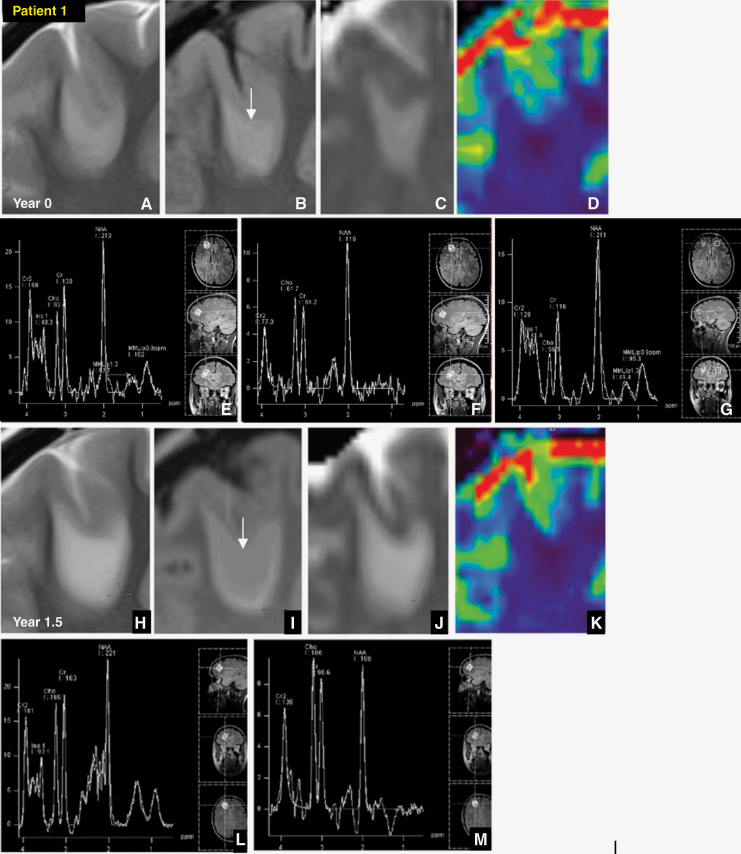
Patient 1, *20-year-old female with 1p19q non-codeleted ATRX mutant p53 overexpressed MGMT 9.75%.* (A) On the index study the non-enhancing tumor in the right frontal lobe showed homogenous T2 hyperintensity, (B, arrowed) without central FLAIR suppression or hyperintense rim, (C) no diffusion restriction on apparent diffusion coefficient (ADC) maps, (D) no raised rCBV, and no enhancement (not shown). (E) Index short TE single-voxel spectroscopy (SVS 30) showed a slight fall in NAA but otherwise normal Cho/Cr, (F) however, early elevation in choline/creatine was more obvious on intermediate TE spectroscopy (SVS 135), (G) with contralateral side for comparison. (H) T2-FLAIR mismatch was only positive at 1.5 years, T2 homogeneous, (I, arrowed) with central FLAIR suppression and peripheral hyperintense rim more evident on 3D FLAIR. (J) ADC, and (K) perfusion maps were unchanged, with only marginal increase in size. (L) NAA continued to show further falls on both SVS 30, and (M) SVS 135 with further mild increase in Cho/Cr, more obvious on SVS 135. The tumor was successfully resected with no recurrence by year 5.5.

7/9 T2-FLAIR mismatch astrocytomas underwent multiparametric MRI. The first three tumors were very small at presentation, all under < 20 × 20 × 20 mm. The earliest and smallest T2-FLAIR mismatch tumor is shown in Figure 2. The lesion did not show T2-FLAIR mismatch sign until the follow-up scan 1.5 years later. A similar pattern was found in case 2, only showing T2-FLAIR mismatch at year 2.5. Case 3 showed T2-FLAIR mismatch at presentation. None of these early tumors showed enhancement, internal diffusion restriction, or raised rCBV > 2. All showed similar metabolic spectra (Figure 2). In all these 3 cases, early Cho/Cr inversion was associated with the development of T2-FLAIR mismatch (Table 1).

**Table 1.** Nine Classic T2-FLAIR Mismatch Astrocytomas, Including Pretreatment Multiparametric Imaging When Available

No.	Sex/ age	Laterality/ lobe	T2/FLAIR margin, shape, T2 signal, enhamcement	rCBV	Cho/Cr	Size (AP × TR × CC)	Treatment/histology/follow-up	1p19q	ATRX	p53	MGMT %	Integrated diagnosis
1	F 20	R FRONTAL	Sharp Rounded T2 Homogeneous Non-enhancing	*Month 0* 0 *Month 10* 0 *year 1 Mo 8* 0	*Month 0* N (SVS 30) N(SVS 135) *Month 10* N (SVS 30) 1.1 (SVS 135) *year 1 Mo 8* N (SVS 30) 1.1 (SVS 135)	*Month 0* 22 × 14 × 27 mm *Month 10* 25 × 15 × 29 mm *year 1 month* *8* 25 × 15 × 29 mm *year 1 month 11* 29 × 19 × 32 mm	No FLAIR suppression No FLAIR suppression Classic T2-FLAIR mismatch Resected year 1 month 11 + radiotherapy and temozolomide Grade 3 Stable at year 5 of follow-up	Non-codeleted	Mut	Overexpressed	9.75	Astro
2	M 43	R FRONTAL	“	*Month 7* 0	*Month 7* N (SVS 30) 1.3 (SVS 135)	*Month 0* 20 × 11 × 20 mm *Month 7* 22 × 13 × 20 mm *Month 16* 26 × 13 × 22 mm	Classic T2-FLAIR mismatch Classic T2-FLAIR mismatch Slow growth. Debulked month 16, completed radiotherapy and PCV Grade 2 Stable on clinical follow-up till year 2.5 then lost to follow-up		Mut	Overexpressed		Astro
3	F 63	R FRONTAL	“	*Month 2* 0	*Month 2* N (SVS 30)	*Month 0* 22 × 20 × 18 mm *Month 2* 22 × 19 × 20 mm *year 1 month5* 28 × 23 × 25 mm *year 3 month3* 30 × 25 × 26 mm	No FLAIR suppression No FLAIR suppression Classic T2-FLAIR mismatch Resected year 3 month 3, adjuvant radiotherapy and temozolomide Grade 3 Stable year 6.5 of follow-up		Mut	Unaltered	9.5	Astro
4	M 43	R FRONTAL	“	*Month 5* 0 *year 1 month 8* 0 *year 2 month 4* 0	*Month 5* 1.37 (SVS 30) 1.53 SVS 135) 2.33 (CSI 30) *year 1 month 8* 1.3 (SVS 30) 1.7 (SVS 135) 1.4 (CSI 135) *year 3 month 4* 1.5 (SVS 30) 1.5 (CSI 135)	*Month 0* 38 × 29 × 43 mm *Month 5* 41 × 30 × 40 mm *year 1 month 8* 51 × 40 × 49 mm *year 2 month 4* 61 × 43 × 56 mm *year 2 month 8* 61 × 48 × 55 mm	Classic T2-FLAIR mismatch Continued to expand and become more internally heterogeneous, showing cystic change Debulked year 2 month 8 + radiotherapy and PCV Grade 2 Mild shrinkage year 6 month 8	Non-codeleted	Mut		0	Astro
5	F 31	R INSULA	“	*year 1 month 9* 0 *year 3 month 10* 0	*year 1 month 9* 1.56 (SVS 30) 1.86 (SVS 135) 1.37 (CSI 30) *year 3 month 10* 1.61 (SVS 30) 2.02 (SVS 135) 2.14 (CSI 30)	*Month 0* 24 × 20 × 28 mm *year 1 Mo1* 31 × 22 × 21 mm *year 1 month 9* 31 × 26 × 37 mm *year 2 month 10* 43 × 34 × 40 mm *year 3 month 10* 47 × 41 × 50 mm *year 4 month 4* 53 × 44 × 55 mm	Classic T2 FLAIR mismatch Tumor expanded, contnuing to show relatively little core heterogeneity or cystic change. Debulked year 4 month 4 + radiotherapy Grade 2 Slow infiltration at margins into temporal lobe by year 7 month 4.		Mut	Unaltered		Astro
6	F 36	L INSULA	“	*Month 10* 0	*Month 0* *Month 10* 1.5 (SVS 30) 1.8 (SVS 135) *year 4 month 10* 1.8 (SVS 30) 2.6 (SVS 135)	*Month 0* 45 × 29 × 28 mm *Month 10* 48 × 31 × 28 mm *year 1 month 10* 49 × 35 × 30 mm *year 4 month 10* 61 × 37 × 32 mm *year 5 month 5* 61 × 37 × 32 mm	Classic T2-FLAIR mismatch. Little expansion but tumor became more internally complex Partial debulking year 1 month 10 Grade 2 Growth of intratumoral nodule year 2 - 5, abnormal spectroscopy preceding enhancement 6 months later. Re-debulking year 5 month 5 + radiotherapy and temozolomide Grade 3	Non-codeleted	Mut	Unaltered	51.25	Astro
7	M 21	L INSULA	“	*Month 0* x 2 foci > 2	*Month 0* 1.61 (SVS 30) Lactate peak	*Month 0* 58 × 36 × 55 mm *Month 3* 59 × 36 × 55 mm	Classic T2-FLAIR mismatch. No significant expansion. Debulked Month 4 + chemoradiotherapy Grade 3 Stable clinical follow-up year 1 month 10		Mut	Overexpressed	11.25	Astro
8	F 30	R TEMPORAL	“				Classic T2-FLAIR mismatch Grade 3		Mut	Overexpressed	38.75	Astro
9	F 32	L FRONTAL	“				Classic T2-FLAIR mismatch Grade 2	Non-codeleted	Mut	Overexpressed	20.25	Astro

All *were* rounded, sharply marginated, T2 homogeneous. rCBV, relative cerebral blood volume on T2* DSC perfusion. SVS, single-voxel spectroscopy at short (30 ms, SVS 30) and intermediate (135 ms, SVS 135) TEs. CSI, chemical shift imaging at short and intermediate TEs (CSI 30, CSI 135).

**Figure 3.**
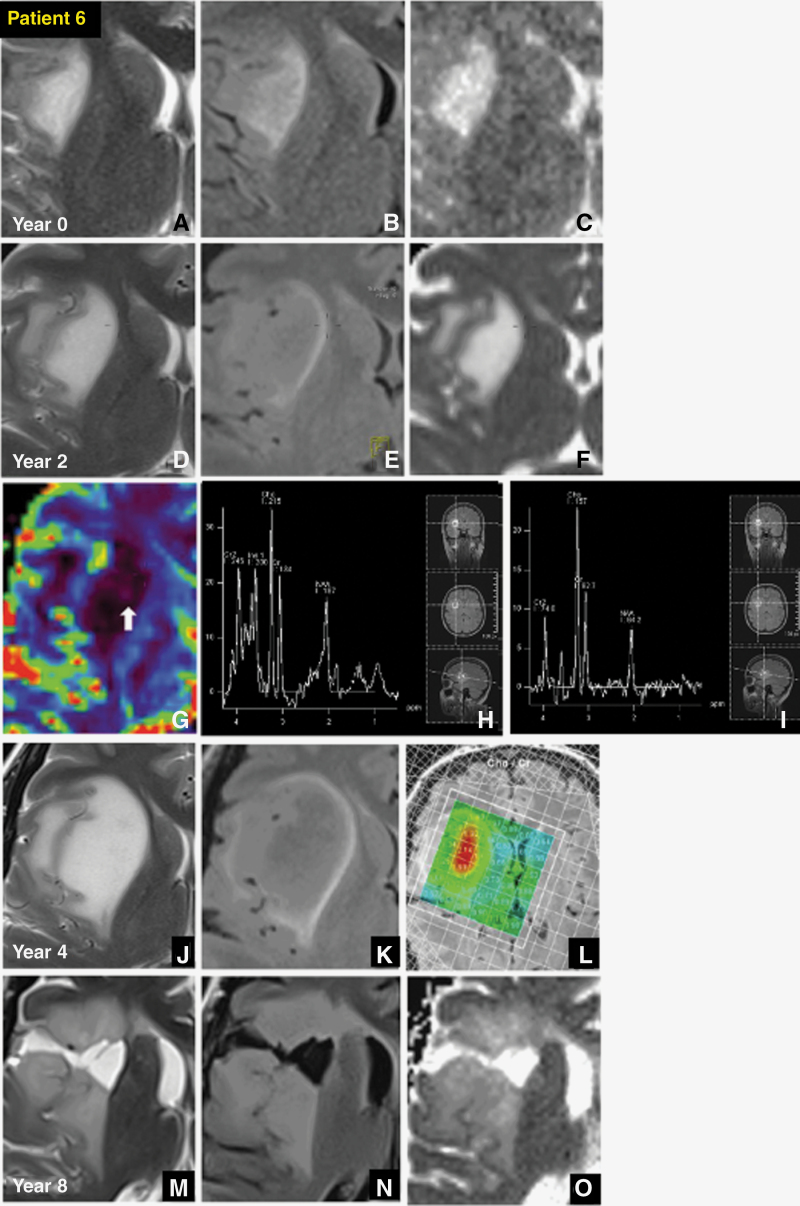
Patient 6, *31-year-old female with ATRX mutant, p53 unaltered, Grade 3 astrocytoma.* On the index study, the non-enhancing tumor in the right insula showed (A) predominantly homogenous T2 hyperintensity, (B) equivocal central FLAIR suppression and hyperintense rim, (C) no diffusion restriction, no high perfusion (not shown). D-F) T2-FLAIR mismatch was positive at year 2, with heterogeneous core on DSC perfusion, but (arrow G) no significantly raised rCBV > 2. Significant falls in NAA were shown on both (H) single-voxel spectroscopy (SVS) 30 and (I) SVS 135 with significant increase in, (H) both NAA/Cho and Cho/Cr up to 1.6 on SVS 30, and (I) significantly raised at 1.9 on SVS 135. (T2, FLAIR; J –K) Tumor increased in size from year 2 to 4, continuing to show T2-FLAIR mismatch without significant core heterogeneity, although (L) core Cho/Cr remained very high > 2 on chemical shift imaging 30. (T2, FLAIR, ADC; M—O) Tumor core was partially debulked year 4 with adjuvant chemoradiotherapy. Disappearance of T2-FLAIR mismatch sign in the post-treatment phase at year 8.

**Figure 4.**
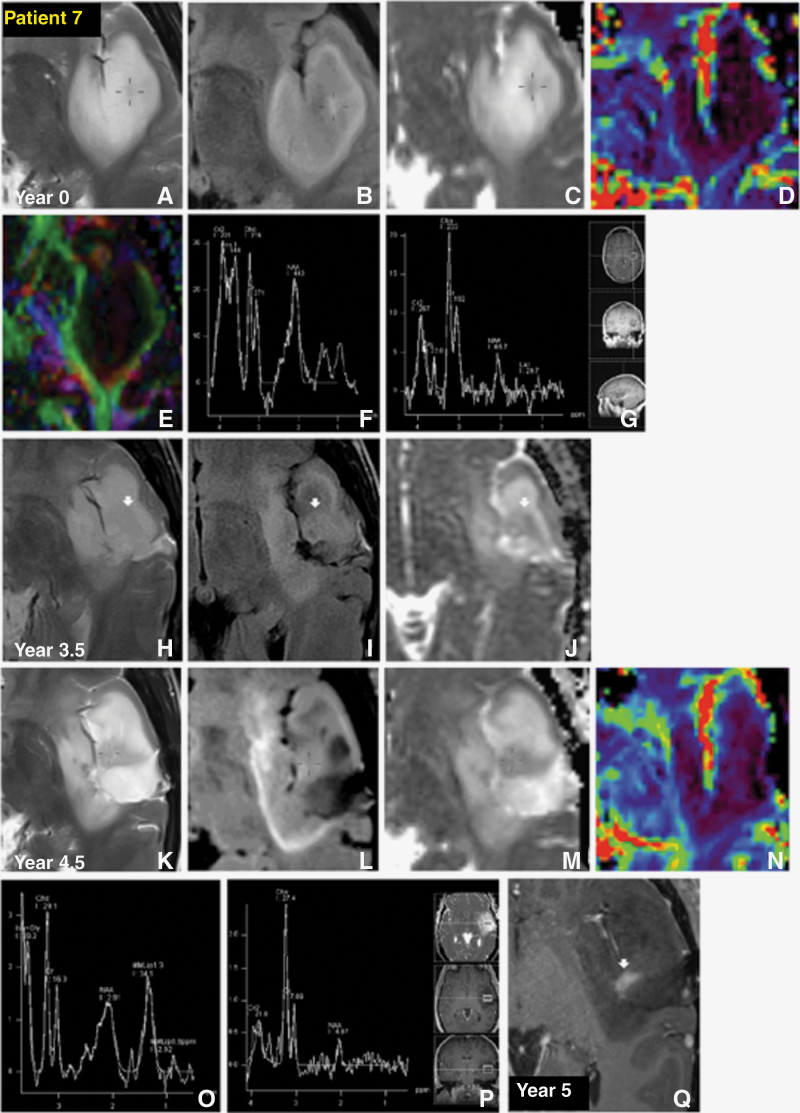
Patient **7** *36-year-old female with 1p19q non-codeleted, ATRX mutant, MGMT 51.25%, Grade 3 astrocytoma.* T2-FLAIR mismatch astrocytoma is shown in the left insula on the index study. (crosshair A) Internal T2 heterogeneity can be seen corresponding to, (crosshair B) areas of non-FLAIR suppression, and (crosshair C) hypointense nodule on apparent diffusion coefficient (ADC) maps, with (D) no high perfusion. (E) External capsule tracts splayed around the lesion on DTI. (F) Falls in NAA evident on single-voxel spectroscopy (SVS) 30, and (G) more so in SVS 135, with (F) moderately raised Cho/Cr at 1.5 on SVS 30, (G) Cho/Cr significantly raised at 1.8 on SVS 135. (H-Q) Although the tumor did not expand significantly it continued to become more internally complex. Partial core debulking at year 1.5 was unsuccessful in removing this nodule, with growing intratumoral residuum seen at the anterior resection margins at year 3.5 on (arrow H—J) T2, FLAIR, and ADC, which continued to (arrow K—M) grow gradually till year 4.5 without (N) increased rCBV. (K-L) This case showed recurrence of T2-FLAIR mismatch in conjunction with recurrent solid disease at the core. (O—P) Significant falls in NAA continued with Cho/Cr remaining very high > 1.8, and progressively increasing on both (O) SVS 30 and (P) SVS 135. (arrow Q) Structural and spectroscopic changes preceded clear enhancement shown 6 months later at year 5 following which the tumor was re-debulked at year 5.5. Histology showed evidence of anaplasia, consistent with transformation to grade 3. The patient had a good response to radiotherapy and temozolomide, and was still alive with size reduction in tumor at year 8.5.

The remaining 4/7 T2-FLAIR mismatch tumors were all larger at presentation. All showed large decreases in NAA, 3/4 significantly raised Cho/Cr > 1.8, and 1/4 showed moderately raised Cho/Cr with raised rCBV > 2 (Table 1). Intratumoral choline mapping showed peak Cho/Cr in specific areas of the core, whereas the FLAIR hyperintense rim of the tumor showed normal Cho/Cr and splayed white matter tracts immediately adjacent; a representative choline map is shown in Figure 3L. Raised Cho/Cr either preceded tumor expansion or correlated with structurally abnormal core areas visible as heterogeneity on T2, FLAIR, and ADC maps (Figures 3 and 4). In addition, two of these cases showed regression of T2-FLAIR mismatch after debulking and chemoradiotherapy (Figure 3K vs. 3N and Figure 4I vs 4L). One case showed recurrence of T2-FLAIR mismatch in conjunction with recurrent solid disease at the core, which preceded florid enhancement (Figure 4Q).

## Discussion

The T2-FLAIR mismatch sign is found to be indicative of IDH mutant 1p19q non-codeleted astrocytomas.^16^ The findings of this study shed light on the dynamic changes of the sign, and intratumoral heterogeneity observed through multiparametric MRI techniques. Our results demonstrated that the T2-FLAIR mismatch sign is absent in small lesions, and emerges at a specific stage of tumor growth. Subsequently, the lesion transitions into a heterogeneous state that can be assessed by MR spectroscopy and DSC perfusion imaging.

An interesting observation in our cohort was the presence of roundness and predominant T2 homogeneity in approximately a quarter of astrocytomas, with or without central FLAIR suppression. We emphasize these features in pre-selecting for astrocytomas, which automatically includes those which will go on to show T2-FLAIR mismatch. Among our cases, simply selecting for roundness and T2 predominant homogeneity excluded almost 95% of oligodendrogliomas. This is consistent with some previous studies, which found that a homogeneous, well-defined glioma is more likely to be IDH-mutant 1p19q non-codeleted, rather than IDH-wild type or 1p19q-codeleted.^17,18^ This may reflect underlying differences in tumor biology and microstructural organization in different subtypes, and needs to be further investigated with a larger sample size.

The smallest astrocytomas may begin as rounded T2 homogeneous lesions without FLAIR suppression. The absence of the T2-FLAIR mismatch in small lesions may indicate a relatively homogeneous tumor microenvironment due to their less advanced stage of growth, characterized by relatively uniform population of neoplastic cells and consistent water content. FLAIR suppression may then become more conspicuous after a few years of follow-up, allowing for a diagnosis of T2-FLAIR mismatch. As T2-FLAIR mismatch develops, the earliest changes detectable on spectroscopy are falls in NAA and early inversion of the Cho/Cr ratio (Figure 2). We confirm that T2-FLAIR mismatch may disappear following chemoradiotherapy (Figures 3 and 4), and then recur with relapse (Figure 4).^9^ T2-FLAIR mismatch is a morphological observation primed to detect an astrocytoma at a specific timepoint of maturation. Given its disappearance with treatment effect and return with relapse, it is also likely to be intimately related to tumor activity.

The larger and more mature T2-FLAIR mismatch tumors developed intratumoral heterogeneity, and showed greater falls in NAA and greater relative elevations of choline. They all showed moderate elevations of Cho/Cr > 1.6 and one showed raised rCBV, consistently demonstrated within regions of the tumor core and not the FLAIR hyperintense rim, likely reflecting an unequal distribution of tumor burden. Elevations in Cho/Cr > 1.8 preceded both tumor expansion without visible core heterogeneity (Figure 3), and also structural progression at the core with subsequent enhancement, the latter by almost six months (Figure 4). These changes may reflect increased cellular proliferation, angiogenesis, and the presence of necrotic or cystic areas, which are characteristic features of high-grade tumors.^19,20^ This was confirmed on re-debulking histology of this area showing transformation from grade 2 to grade 3. Our previous study found that when the T2/FLAIR mismatch sign is combined with T2* DSC-perfusion, the histological diagnosis and grading of IDH-mutant, 1p/19q non-co-deleted astrocytoma could be predicted with an accuracy of 100%.^13^ MR spectroscopy and perfusion imaging reveal diverse metabolic and angiogenetic profiles within the tumor, indicative of varying cellular activity and microvascular characteristics.^21^ The observed heterogeneity may arise from spatially distinct regions of necrosis, hypoxia, or angiogenesis within the tumor mass, reflecting its dynamic and multifaceted nature.^22,23^ These abnormal areas with the heaviest tumor burden should be targeted for biopsy or selective debulking.

Therefore, the T2-FLAIR mismatch sign and the subsequent transition towards a heterogeneous state of astrocytoma lesions may signify the progression of the tumors towards higher grades. This finding enables us to non-invasively predict the likelihood of transformation at an early stage, facilitating prompt appropriate clinical intervention.

Furthermore, the utilization of advanced MRI techniques to assess tumor heterogeneity provides valuable imaging guidance for more accurate localization of biopsy target, and aids in precise and selective resection, especially when the tumors are located in challenging anatomical regions. This thereby improves the diagnosis and treatment strategies, and ultimately leads to better outcomes and quality of life for the patients.

The study has several limitations, primarily stemming from its small sample size, which is attributed to the relatively low incidence of the T2-FLAIR mismatch sign and strict inclusion criteria. This study primarily serves as a descriptive analysis rather than an investigation into underlying mechanisms. A larger sample size with prospective design would be necessary for a more comprehensive examination of the mechanisms driving the emergence and disappearance of the T2-FLAIR mismatch sign in astrocytomas.

## Conclusion

The T2-FLAIR mismatch sign is indicative of IDH-mutant 1p19q non-codeleted astrocytomas. The sign is absent in small lesions and emerges at a specific stage of tumor growth. By assessing the subsequent heterogeneity, MR spectroscopy and perfusion imaging are able to predict the progression of the tumor toward higher grade. The findings enable noninvasive prediction of the likelihood of malignant transformation at an early stage, and facilitate timely appropriate clinical intervention. This study also suggests the potential diagnostic value of “roundness” as a marker for identifying astrocytomas at an early stage, warranting further research into its diagnostic utility.

## Data Availability

Data will be made available upon request.
